# Letter to Editor: In response to existence of SARS-CoV-2 in the peritoneal fluid

**DOI:** 10.61622/rbgo/2024rbgo24

**Published:** 2024-04-09

**Authors:** Gustavo Romero-Velez, Guillermo Ponce de Leon-Ballesteros, Juan Barajas-Gamboa, Jerry Dang, Andrew Strong, Mathew Kroh

**Affiliations:** 1 United States of America Endocrine and Metabolism Institute Cleveland Clinic Cleveland OH America Endocrine and Metabolism Institute, Cleveland Clinic, Cleveland, OH, United States of America.; 2 Hospital Angeles Morelia Department of Surgery Morelia Mexico Department of Surgery, Hospital Angeles Morelia, Morelia, Mexico.; 3 United Arab Emirates Cleveland Clinic Abu Dhabi Digestive Disease Institute America Digestive Disease Institute, Cleveland Clinic Abu Dhabi, United Arab Emirates; 4 United States of America Digestive Disease and Surgery Institute Cleveland Clinic Cleveland OH America Digestive Disease and Surgery Institute, Cleveland Clinic, Cleveland, OH, United States of America.

We read with great interest the article recently published by Ilgen et al.^(1)^ in which the authors prospectively collected peritoneal samples of patients positive for SARS-CoV-2 undergoing cesarean section. Their study increases the understanding of SARS-CoV-2 and the risk of infection while performing surgery, however, we would like to caution the authors in their conclusion. The authors findings are that none of the eight patients were positive for SARS-CoV-2 in their peritoneal samples and thus conclude that the exposure due to aerosolization of surgical fumes does not seem to be likely. Although the majority of case reports and case series have not been able to identify the presence of SARS-CoV-2 in the peritoneum, our recent systematic review on the topic found that 5.9% of the cases are likely to be positive, which is even higher for patients with severe disease.^(2^the virus responsible for COVID-19, in the abdominal cavity as well as in other abdominal tissues which surgeons are exposed has been investigated in several studies. The aim of the present systematic review was to analyze if the virus can be identify in the abdominal cavity.\nMethods We performed a systematic review to identify relevant studies regarding the presence of SARS-CoV-2 in abdominal tissues or fluids. Number of patients included as well as patient's characteristics, type of procedures, samples and number of positive samples were analyzed.\nResults A total of 36 studies were included (18 case series and 18 case reports) Thus, there is a theoretical risk of exposure to healthcare providers while performing surgery in infected patients. In a similar way, the Society of American Gastrointestinal and Endoscopic Surgeons (SAGES) guidelines in the topic recommends protective measures to prevent the exposure in the era of COVID-19.^(3)^ or suspecting of having, the SARS-CoV-2 as well as the healthcare team involved.\nMethods Systematic literature reviews were conducted for 2 key questions (KQ^)^
