# Supplementary material for: Letter to Editor: In response to existence of SARS-CoV-2 in the peritoneal fluid
Source: Rev Bras Ginecol Obstet. 2024 Apr 9;46:e-rbgo24. doi: 10.61622/rbgo/2024rbgo24 (PMC11075428; doi:10.61622/rbgo/2024rbgo24)
Supplement: Related texts

# Reply to: Existence of SARS-CoV-2 in the peritoneal fluid

**Authors:** Orkun Ilgen, Mehmet Eyuphan Ozgozen, Ozgur Appak, Begum Ertan, Hikmet Tunc Tımur, Omer Erbil Dogan, Cemal Posacı

**Affiliations:** 1 Dokuz Eylul University School of Medicine Department of Obstetrics and Gynecology Izmir Turkey Department of Obstetrics and Gynecology, School of Medicine, Dokuz Eylul University, Izmir, Turkey.

We are gratified to note the international interest our article has sparked among medical practitioners. Romero-Velez et al.^(1)^ recently presented an article entitled "Presence of SARS-CoV-2 in abdominal tissues and biologic fluids during abdominal surgery: a systematic review"; highlighting the substantial risk of viral particle dispersion during abdominal surgery, particularly in severe disease cases. Our research focuses on cases from the earlier stages of the pandemic when our understanding of the virus was not as advanced as it is today. Another Italian study by Fabbri et al.^(2)^ stated that all their samples tested negative for SARS-CoV-2 RNA. Still, like other researchers, they emphasized that the risk could not be entirely ruled out and advised appropriate precautions. We concur that further research is necessary to shed more light on this issue.^(3)^ Our primary goal should be to minimize the surgical team exposure to surgical fumes and other patient-derived substances, regardless of the specific pathogen. SARS-CoV-2 has undoubtedly brought more attention to this concern. We align ourselves with the guidelines of the Society of American Gastrointestinal and

Endoscopic Surgeons (SAGES)^(4)^ and recommend implementing protective measures, even though numerous studies have failed to detect the virus in peritoneal samples. We extend our gratitude once more to Romero-Velez et al.^(1)^ for maintaining the relevance of this discussion.

## References

[1] Romero-Velez G, Ponce de Leon-Ballesteros G, Al Zubaidi M, Barajas-Gamboa JS, Dang J, Corcelles R (2023). Presence of SARS- CoV-2 in abdominal tissues and biologic fluids during abdominal surgery: a systematic review. Surg Endosc.

[2] Fabbri N, Pesce A, Ussia A, D’Urbano F, Pizzicotti S, Greco S (202). Swab test in biological fluids as predictor of COVID-19 transmission risk during surgery: a prospective cross-sectional study from an Italian COVID center. BMC Surg.

[3] Ilgen O, Ozgozen ME, Appak O, Ertan B, Tlmur HT, Dogan OE (2023). Existence of SARS-Cov-2 in the peritoneal fluid. Rev Bras Ginecol Obstet.

[4] Collings AT, Jeyarajah DR, Hanna NM, Dort J, Tsuda S, Nepal P (2022). SAGES 2022 guidelines regarding the use of laparoscopy in the era of COVID-19. Surg Endosc.
